# Surpoids et obésité dans la population générale de 5 à 19 ans en milieu urbain bamakois (Mali)

**DOI:** 10.11604/pamj.2014.19.351.4380

**Published:** 2014-12-03

**Authors:** Hamidou Oumar Bâ, Ichaka Menta, Youssouf Camara, Plutôt Seydou Doumbia, Mamadou Bocary Diarra

**Affiliations:** 1 Centre Hospitalier Universitaire Gabriel Touré, Bamako, Mali; 2 Centre Hospitalier Universitaire Kati, Bamako, Mali; 3 DER de Santé Publique-Faculté de Médecine et d'Odontostomatologie, Bamako, Mali; 4 Centre Hospitalier Mère-Enfant Le Luxembourg, Bamako, Mali

**Keywords:** Surpoids, obésité, enfants, adolescents, population générale, Bamako, Overweight, obesity, children, adolescents, general population, Bamako

## Abstract

**Introduction:**

Déterminer la prévalence du surpoids et de l'obésité dans la population âgée de 5 à 19 ans et fournir des données de référence pour de futures études.

**Méthodes:**

Notre échantillon est issu de la première étude sur les pathologies cardiovasculaires basée sur l'approche STEP de l'Organisation Mondiale de la Santé (OMS) en sélectionnant tous les sujets âgés de 5 à 19 ans. Nous avons utilisé les méthodes de l'OMS et de l'International Obesity Task Force (IOTF) pour déterminer la prévalence du surpoids et de l'obésité dans la population générale.

**Résultats:**

La moyenne d’âge était de 11,75 ans ± 4,387 et le sex-ratio M:F était de 0,79. les moyennes pour le poids et la taille étaient de 36,85 kg et 143,48 cm. Selon les critères OMS 1,61% des garçons et 3,28% des filles étaient en surpoids et 0,92% des garçons contre 1,46% des filles obèses. Selon les critères de l'IOTF 4,10% des garçons et 5,94% des filles étaient en surpoids tandis que 0,72% des garçons et 2,68% des filles étaient obèses.

**Conclusion:**

Malgré sa faible prévalence le surpoids et l'obésité doivent être régulièrement étudiés pour reconnaître des tendances et prendre des mesures adéquates de prévention. Les 2 méthodes utilisées ont permis d'avoir des données de référence pour de futures études au Mali et ailleurs.

## Introduction

Le surpoids (SP) et l'OB sont reconnus depuis longtemps comme problème de santé publique avec plus d'1/4 milliards d'adultes en surpoids et 500 millions d'obèses en 2008 avec des prévisions de 2,3 milliards d'adultes en surpoids et 700 millions d'obèses en 2015 [1]. Il a été note une prévalence accrue de l'obésité de l’âge préscolaire à l'adolescence dans le monde [2–7]. La plupart des études étaient nord-américaines ou européennes jusqu'aux travaux de Cole et al ayant abouti à l’établissement de seuils pour l'IMC chez les enfants et adolescents de 2 à 18 ans en 2000 [3] et de l'Organisation Mondiale de la Santé (OMS) en 2006 avec des normes basées sur les déviations standards de l'IMC pour la tranche d’âge 5 à 19 ans [8]. Ces deux méthodes sont les plus utilisées actuellement. Les données de prévalence sur le SP et l'OB sont rares en Afrique, particulièrement en Afrique sub-saharienne, le Mali ne faisant pas exception. Pour remédier au manqué de données, nous avons initié cette étude en reprenant les données de l'enquête sur les pathologies cardiovasculaires dans le district de Bamako en appliquant les 2 méthodes sus-citées [3]. L'objectif du travail était de déterminer la prévalence du SP et de l'OB dans la population générale de 5 à 19 ans et fournir des données de références pour de futures études.

## Méthodes

Notre échantillon est issu de la première enquête basée sur l'approche STEP de l'OMS et conduite dans le district de Bamako par l’équipe du Pr Touré.

### Sélection des patients

Tous les sujets de 5 à 19 ans de l'enquête initiale ont été inclus dans notre étude (984 enfants et adolescents). L’âge, le sexe, la taille, le poids étant disponibles, nous avons pu utiliser un programme SPSS fourni par l'OMS permettant d'obtenir différents Z-scores notamment celui de l'IMC pour chaque sujet [9].

Définition des termes:

### Références IOTF

Le SP et l'OB ont été définis conformément aux valeurs établies par Cole et al [3].

### Références OMS 2007

Les définitions suivantes basées sur le z-score de l'IMC ont été adoptées [8, 10]: >1 and < = 2 SD: risque de surpoids (RSP); > 2 DS and < = 3SD: surpoids (SP); > + 3 SD: obésité (OB).

### Traitement des données

Le logiciel SPSS dans sa version 12 a été utilisé pour l'analyse des données.

## Résultats

La taille de l’échantillon était de 984 enfants et adolescents de 5 à 19 ans (moyenne 11,75 ± 4,387 ans) avec 55,69% des sujets étaient de sexe féminin. Les enfants de 5 ans étaient les plus représentés dans l’échantillon (Tableau 1). Les moyennes pour le poids et la taille étaient resp. de 36,85 kg (11-105) et 143,48 cm (95-193).

**Tableau 1:** Répartition de l’échantillon par âge et sexe

Age (years)	Sexe		Total
	**M**	**F**	
5	38	46	84
6	47	29	76
7	29	30	59
8	39	37	76
9	18	33	51
10	23	41	64
11	30	37	67
12	40	39	79
13	23	25	48
14	23	35	58
15	28	41	69
16	19	43	62
17	28	38	66
18	30	48	78
19	21	26	47
**Total**	**436**	**548**	**984**

### Prévalences selon les critères OMS (Tableau 2)

Le RSP, le SP et l'OB étaient retrouvés dans resp. 5,5%, 2,6% and 0,3% de l’échantillon. Le RSP était le plus retrouvé pour les 5 et 6 ans (10,53% and 10,64%) chez les garçons et 12,50% chez les filles. La plus forte prévalence de SP était de 6,90% chez les garçons et 9,3% chez les filles resp. Pour les 7 et 16 ans. Les prévalences pour l'OB étaient de 7,14% chez les garçons et 6,67% chez les filles pour les 15 et 7 ans.

**Tableau 2:** Répartition par âge et sexe du SP et de l'OB en% selon les critères OMS [8, 9] dans la population générale de 5 à 19 ans dans la ville de Bamako

Age	NUW		RSP		SP		OB		Effectif	
	M	F	M	F	M	F	M	F	M	F
**5**	1	8	3	2,17	2,63	2,17	2,63	2,17	38	46
**6**	89,3	86,2	10,6	3,45	0,00	6,90	0,00	3,45	47	29
**7**	89,6	90,0	3,45	3,33	6,90	0,00	0,00	6,67	29	30
**8**	92,3	89,1	5,13	5,41	2,56	5,41	0,00	0,00	39	37
**9**	88,8	93,9	0,00	3,03	5,56	0,00	5,56	3,03	18	33
**10**	95,6	82,9	4,35	4,88	0,00	7,32	0,00	4,88	23	41
**11**	90,0	97,3	10,0	2,70	0,00	0,00	0,00	0,00	30	37
**12**	92,5	94,8	7,50	0,00	0,00	5,13	0,00	0,00	40	39
**13**	95,6	84,0	4,35	12,0	0,00	4,00	0,00	0,00	23	25
**14**	95,6	94,2	4,35	0,00	0,00	2,86	0,00	2,86	23	35
**15**	92,8	92,6	0,00	7,32	0,00	0,00	7,14	0,00	28	41
**16**	94,7	79,0	0,00	11,6	5,26	9,30	0,00	0,00	19	43
**17**	96,4	92,1	0,00	5,26	3,57	2,63	0,00	0,00	28	38
**18**	93,3	85,4	6,67	12,5	0,00	2,08	0,00	0,00	30	48
**19**	100	88,4	0,00	11,5	0,00	0,00	0,00	0,00	21	26
**Total**	92,2	89,6	5,28	5,66	1,61	3,28	0,92	1,46	436	548

NUW: Normal ou déficit pondéral RSP: risque de surpoids SP: Surpoids OB: obésité

### Prévalences selon l'IOTF (Tableau 3)

La prévalence du SP et de l'OB était de 5,12% et 1,81%. La plus forte prévalence du SP était de 10,34% chez les garçons et de 16,28% chez les filles resp. pour les sujets de 7 et 16 ans. Pour l'OB la plus forte prévalence était de 5,56% chez les garçons et de 7,32% chez les filles resp. Pour les 9 et 10 ans.

**Tableau 3:** Répartition par âge et sexe du SP et de l'OB en% selon les critères IOTF [3] dans la population générale de 5 à 19 ans dans la ville de Bamako

Age	NUW		OW		OB		N	
	M	F	M	F	M	F	M	F
**5**	92,1	95,6	7,89	2,17	0,00	2,17	38	46
**6**	93,6	89,6	6,38	3,45	0,00	6,90	47	29
**7**	89,6	96,6	10,3	0,00	0,00	3,33	29	30
**8**	94,8	89,1	5,13	8,11	0,00	2,70	39	37
**9**	88,8	96,9	5,56	3,03	5,56	0,00	18	33
**10**	100	85,3	0,00	7,32	0,00	7,32	23	41
**11**	100	97,3	0,00	2,70	0,00	0,00	30	37
**12**	95,0	94,8	5,00	2,56	0,00	2,56	40	39
**13**	100	88,0	0,00	12,0	0,00	0,00	23	25
**14**	100	94,2	0,00	0,00	0,00	5,71	23	35
**15**	96,4	95,1	0,00	4,88	3,57	0,00	28	41
**16**	94,7	81,4	5,26	16,2	0,00	2,33	19	43
**17**	96,4	92,1	0,00	5,26	3,57	2,63	28	38
**18**	93,3	85,4	6,67	12,5	0,00	2,08	30	48
**Total**	95,1	91,3	4,10	5,94	0,72	2,68	415	522

NUW: Normal ou déficit pondéral RSP: risque de surpoids SP: Surpoids OB: obésité

## Discussion

L’étude conçue selon l'approche STEP a été réalisée sur un échantillon représentatif de la population générale à Bamako. Des études similaires sont rares dans les pays africains en particulier au Sud de Sahara. À notre connaissance des études regroupant à la fois un échantillon de 5 jusqu’à 19 ans et ayant utilisé les méthodes OMS et IOTF sont inexistantes. Cela rend la comparaison des données difficile. Notre objectif était essentiellement de fournir des données pouvant être utilisées comme références à de futures études. L’étude a permis de faire les constats suivants: les prévalences obtenues sont inférieures à celles des études de pays développés, même si les définitions dans ces études étaient basées sur les percentiles [1–13]; les prévalences obtenues en utilisant la méthode de l'IOTF sont plus élevées que celles obtenues par la méthode de l'OMS; quelle que soit la méthode utilisée (OMS, IOTF), les prévalences pour le SP sont élevées chez les filles que chez les garçons. De même l'OB tend à disparaître entre 10 et 14 ans chez les garçons contrairement aux filles; la prévalence de l'OB était plus élevée chez les filles Notre étude a souffert de certains manques, en particulier la non-disponibilité des analyses biologiques (essentiellement pour raison financière) et aussi l'impossibilité de vérifier dans l'ascendance le SP et l'OB.

## Conclusion

Malgré sa faible prévalence le surpoids et l'obésité doivent être régulièrement étudiés pour reconnaître des tendances et prendre des mesures adéquates de prévention. Les 2 méthodes utilisées ont permis d'avoir des données de référence pour de futures études au Mali et ailleurs.
